# The Medical News Visiting List

**Published:** 1885-12-20

**Authors:** 


THE MEDICAL NEWS VISITING LIST.—A complete pocket-book of useful memoranda for physicians and surgeons, with blanks suitable for keeping the professional and business records of a practice aggregating thirty patients per day. Wallet form, handsome red seal binding, tucks, pocket, pencil and rubber, $1. With patent thumb-letter index for rapid use, 25 cents additional.

